# Strengthening Medical Product Regulation in Low- and Middle-Income Countries

**DOI:** 10.1371/journal.pmed.1001327

**Published:** 2012-10-23

**Authors:** Charles Preston, Mary Lou Valdez, Katherine Bond

**Affiliations:** Office of International Programs, US Food and Drug Administration (FDA), Silver Spring, Maryland, United States of America

## Abstract

Charles Preston and colleagues use case studies to describe how regulatory systems for medical products and medicines must be strengthened in low and middle income countries to assure adequate quality, safety and efficacy.

**Summary Points**

- Few global initiatives focus on strengthening low- and middle-income country medical product regulatory systems.
- However, globalization and the scaling up of medicines and vaccines to the developing world are highlighting the urgent need for systems to assure product efficacy, safety, and quality.
- This article explores case studies in regulatory domains such as global product supply chains, clinical trials, premarket approval, post-market surveillance, and regulatory science to demonstrate the essential value of medical product regulatory systems to low- and middle-income countries.
- Here, a viable path is put forward for making this important topic a global health priority.

## Introduction

Medical product regulatory systems are central to health systems; they ensure high quality and safe interventions like drugs, vaccines, and medical devices for patients who need and count on them. The World Health Organization (WHO) recognizes this fact and includes regulatory system functions as one of the six core building blocks of health systems: access to medical products, vaccines, and technologies of assured quality, safety, and efficacy [1].

Although WHO has recognized their importance, to date, little attention has been focused on regulatory systems in low- and middle-income countries. They have not featured prominently in global health and development assistance programs, and few strategic documents of major global health initiatives, including the United States Global Health Initiative, reference regulatory systems [2].

The global activities that do involve regulatory systems typically involve high-income countries. For example, the International Conference on Harmonization of Technical Requirements for Registration of Pharmaceuticals for Human Use (ICH), which harmonizes regulatory standards and processes for the pharmaceutical industry, includes regulatory authorities from the European Union, Japan, and the United States [3]. The membership of the International Medical Device Regulators Forum is similarly comprised, including Australia, Canada, the European Union, Japan, and the United States. Brazil is the only low- or middle-income country that is a member [4].

The lack of attention to medical product regulatory systems in low- and middle-income countries is a significant gap that needs to be bridged.

## Proposal

We propose that strengthening regulatory systems in low- and middle-income countries must become a global health priority, and explain the imperative in terms of globalization and the rapid scale up of medicines to the developing world. Here, we explore case studies from key regulatory domains, for example, product supply chains, clinical trials, pre-market approval, post-market surveillance, and regulatory science to show the multiple ways that strengthening these systems can contribute to global health.

### Case Studies

One reason low- and middle-income country regulatory systems have not traditionally received development assistance is that these systems did not manufacture products for high-income country markets. However, globalization is dramatically changing that fact. Now, 30% of both drugs and medical devices used in the United States come from abroad [5]. Product supply chains are global, and the paths they take typically originate in, or weave through, low- and middle-income countries.

The case of tainted cough syrup in Panama is an excellent example. When an imitation sweetening ingredient from China arrived in Panama in 2006, government pharmacists unknowingly mixed it into 260,000 bottles of cold medicine [6]. The result was exposure to a deadly chemical commonly used in antifreeze, diethylene-glycol. Although estimates continue to be revised, it caused well over 100 deaths and many injuries [7]. The poisonous shipment traveled across continents, from Asia to Europe to the Americas, and at each step of the way, eluded detection—even though the original product name included initials derived from a Chinese word meaning “substitute” [6]. Worse, the manufacturer was not authorized to make pharmaceutical ingredients, but this fact went undiscovered because the certificate attesting to the product's purity was stripped of the name of the manufacturer [6]. The case shows the increasing complexity, and risk, of global product supply chains. More importantly, it highlights the deep impact of globalization on regulatory systems.

Indeed, regulators everywhere must contend with this new force. At the FDA, for example, its domestic public health mission can now only be accomplished by operating within a global context. In 2008, it began to establish posts in strategic locations around the world, and recently embarked on a new strategy called the Pathway to Global Product Safety and Quality [8], which envisions a global product safety net and emphasizes partnerships like global coalitions and leveraging public and private third parties. Other regulatory authorities are adjusting to the imperatives of globalization too, especially as clinical trials [9] and manufacturing [10] move to their shores. However, many of these agencies were poorly resourced before, and now because of globalization, are being stressed even further.

An example of this is in Africa, where countries are increasingly the target of vaccine clinical trials for HIV, malaria, and other infectious diseases. Yet in 2005, the WHO identified gaps in the functioning of many African regulatory systems, such as the lack of legal frameworks, regulatory standards and guidance, and the training/recruitment/retention of regulatory experts and professionals, to oversee these trials [11]. As a result, an initiative was begun to provide critical expertise on clinical trials regulation—the African Vaccine Regulatory Forum (AVAREF) [12]. WHO, Health Canada, the European Medicines Agency, and FDA participate as expert advisors, and to date, AVAREF has enhanced communication between regulators, encouraged the adoption of model regulatory procedures, and spurred several countries to adopt Good Clinical Practice inspections [13]. It has also fostered the development of a regional strategy and the formation of new NGOs dedicated to funding regulatory capacity building [13].

Globalization is not the only force driving the need to strengthen regulatory systems in low- and middle-income countries. The explosion in funding for global health is also a factor. As programs like the US President's Emergency Plan for AIDS relief (PEPFAR) provide anti-retrovirals (ARVs) to millions of people, it is necessary to ensure that the medications are safe and of high quality. Systems in many low- and middle-income countries are not equipped to perform such assurance functions, and in recognition of this fact, WHO set up a prequalification program to certify medicines for priority diseases and guide countries and procurement agencies on their purchasing of products [14].

In the case of PEPFAR, the US FDA used an established mechanism called “tentative approval” to assure safety, quality, and efficacy. Through it, FDA reviews generic and new drug combinations for purchase by the PEPFAR program. Because many ARVs are currently on patent in the United States, this mechanism allows products to be developed for use in AIDS stricken countries, even if they are still on patent. The generic versions tentatively approved have the effect of decreasing overall drug costs, and in fact, from 2005–2008, the mean PEPFAR purchase price for the ten highest volume ARV formulations declined an average of 42% under tentative approval, and by as much as 86% [15]. This saved the Plan US$380 million during a three-year period [15]—enough to treat 200,000 more people [16]. Earlier in the decade, the typical person could expect to spend around US$10,000 per year; now the cost has come down to under US$100 per person per year for many regimens [17].

As of August 2012, the FDA has found 152 different medications to meet its manufacturing quality and bioequivalence standards. However, it is important to recognize that PEPFAR's programmatic strategy is country ownership [18], and in the future, this implies that national and regional regulatory systems rather than FDA will be responsible for assurance activities, such as dossier assessment and inspections for quality standards. Will they be prepared to perform this function?

The corollary to assuring quality and safety before market authorization is to do this after approval as well. However, many low- and middle-income countries lack the regulatory capacity to undertake post-market surveillance—especially those in sub-Saharan Africa, where many of the drugs and vaccines are sent. Research funded by an interagency agreement between FDA and the US Agency for International Development shows that the vast majority of these countries are not able to collect information on the safety or quality of products, or take regulatory action once a problem has been identified [19]. When death and injury result, such as 3,000 deaths from inoculation with counterfeit meningitis vaccine in Niger in 2005 [19], patients lose confidence in their medical products. This attitude could lead to poor adherence and antimicrobial resistance, to reduced demand for treatments, and to inappropriate switching to more toxic or ineffective therapies [20]. More alarming is the fact that these cases are only the tip of the iceberg. Without adequate surveillance, most death and disease resulting from unsafe medicine is not detected. Donors like the Global Fund to Fight AIDS, TB and Malaria (Global Fund), the GAVI Alliance, and the Bill and Melinda Gates Foundation are beginning to support post-market surveillance work, but much more needs to be done.

Another example of why strengthening regulatory systems in low- and middle-income countries is important to global health is in the area of regulatory science, which is the development of public sector tools, methods, and models to accelerate and improve the regulation of innovative and generic products. With the goal of increasing access to medicines, FDA scientists invented a method for conjugating meningitis vaccine that could easily be adopted by a low- or middle-income country production facility. They signed a technology transfer agreement with the Gates Foundation, WHO, and the Program for Appropriate Technology in Health, and trained scientists from industry and government in the conjugation methodology. FDA scientists continued to share their technical expertise as the vaccine went through clinical trials, and in December of 2009, the Drugs Controller General of India licensed the new vaccine, called MenAfriVac. One year later, mass immunizations were launched in Africa's meningitis belt—at the low cost of US$0.50 per dose [21]. To date, 20 million people have been vaccinated [22]. If regulatory science can be strengthened in low- and middle-income countries, many more successes like MenAfriVac will be possible.

## Challenges to Implementation

The global health community is gradually awakening to the role that regulatory systems play in low- and middle-income countries, as evidenced by the case studies in this paper, but more needs to be done to make strengthening these systems a global health priority.

Efforts must be targeted and prioritized, with an end goal of sustainability in mind. It is not necessary for every low- and middle-income country system to be equivalent to that of a stringent authority like Canada or the European Union. Rather, there are several foundational elements that all regulatory systems, rich or poor, should have. The US Institute of Medicine (IOM) identified three minimal capabilities: (1) a rule-making process that allows all stakeholders to comment on proposed regulations; (2) a protocol for different regulatory agencies to share information and oversight along supply chains; and (3) a method to identify when regulatory actions are necessary [23].

Much more thinking needs to occur on what the IOM's suggested core capacities mean and how they might be implemented. To this end, it will be important to begin a global dialogue on the subject of regulatory system strengthening in low- and middle-income countries. Various initiatives need to be coordinated and leveraged, including those already underway, such as the African Medicines Regulatory Harmonization initiative [24], which seeks to speed medical product registrations, the Pan American Health Organization's Regional Platform on Access and Innovation for Health Technologies [25], and the call for a single African medicines regulatory agency by stakeholders such as the African Union and United Nations AIDS [26],[27].

Also, as other global health initiatives, such as the GAVI Alliance, the Global Fund, and the United States Global Health Initiative, turn their efforts towards health system strengthening [28], it is critical that regulatory system strengthening be included as well.

Garnering support for this agenda is not without its challenges. There are numerous competing priorities for political and financial support, especially in the wake of the global financial crisis. Further, it is unlikely that global health will enjoy the same level of resourcing as in the previous decade [29]. In the face of these challenges, it will be important to continue to communicate the value of regulatory systems to global health; to target their strengthening for sustainability; and to coordinate and leverage existing and planned capacity building initiatives. A global dialogue is beginning. Discussions of expanded market access for exports, increased trade opportunities emanating from science-based regulation and regulatory coherence, and more sustainable economic development, should also be included, as they will provide further incentives for countries to come to the table.

## Summary

In summary, the case studies exploring global product supply chains and diethylene glycol poisoning in Panama, clinical trials regulation through AVAREF, premarket assurance through PEPFAR tentative approval, post-market surveillance in sub-Saharan Africa through research on drug and vaccine safety systems, and regulatory science through the creation of a low-cost meningitis vaccine for low- and middle-income countries, demonstrate the essential value of regulatory systems to low- and middle-income countries. When they work, people live; when they fail, people die. As the challenges of globalization mount, and efforts to provide medical products to low- and middle-income countries scale up, there is no better time to put regulatory system strengthening squarely on the global health and development agenda.
